# Millimeter-Sized Marine Plastics: A New Pelagic Habitat for Microorganisms and Invertebrates

**DOI:** 10.1371/journal.pone.0100289

**Published:** 2014-06-18

**Authors:** Julia Reisser, Jeremy Shaw, Gustaaf Hallegraeff, Maira Proietti, David K. A. Barnes, Michele Thums, Chris Wilcox, Britta Denise Hardesty, Charitha Pattiaratchi

**Affiliations:** 1 School of Environmental Systems Engineering, University of Western Australia, Perth, Australia; 2 Oceans Institute, University of Western Australia, Perth, Australia; 3 Wealth from Oceans Flagship, Commonwealth Scientific and Industrial Research Organisation, Perth, Australia; 4 Centre for Microscopy, Characterisation and Analysis, University of Western Australia, Perth, Australia; 5 Institute for Marine and Antarctic Studies, University of Tasmania, Hobart, Australia; 6 Instituto de Oceanografia, Universidade Federal do Rio Grande, Rio Grande, Brazil; 7 British Antarctic Survey, Natural Environment Research Council, Cambridge, United Kingdom; 8 Australian Institute of Marine Science, The UWA Oceans Institute, Perth, Australia; 9 Marine and Atmospheric Research, Commonwealth Scientific and Industrial Research Organisation, Hobart, Australia; Stazione Zoologica Anton Dohrn, Naples, Italy

## Abstract

Millimeter-sized plastics are abundant in most marine surface waters, and known to carry fouling organisms that potentially play key roles in the fate and ecological impacts of plastic pollution. In this study we used scanning electron microscopy to characterize biodiversity of organisms on the surface of 68 small floating plastics (length range = 1.7–24.3 mm, median = 3.2 mm) from Australia-wide coastal and oceanic, tropical to temperate sample collections. Diatoms were the most diverse group of plastic colonizers, represented by 14 genera. We also recorded ‘epiplastic’ coccolithophores (7 genera), bryozoans, barnacles (*Lepas* spp.), a dinoflagellate (*Ceratium*), an isopod (Asellota), a marine worm, marine insect eggs (*Halobates* sp.), as well as rounded, elongated, and spiral cells putatively identified as bacteria, cyanobacteria, and fungi. Furthermore, we observed a variety of plastic surface microtextures, including pits and grooves conforming to the shape of microorganisms, suggesting that biota may play an important role in plastic degradation. This study highlights how anthropogenic millimeter-sized polymers have created a new pelagic habitat for microorganisms and invertebrates. The ecological ramifications of this phenomenon for marine organism dispersal, ocean productivity, and biotransfer of plastic-associated pollutants, remains to be elucidated.

## Introduction

Millimeter-sized plastics resulting from the disintegration of synthetic products (known as ‘microplastics’ if smaller than 5 mm) are abundant and widespread at the sea surface [1]–[7]. These small marine plastics are a toxic hazard to food webs since they can contain harmful compounds from the manufacturing process (e.g. Bisphenol A), as well as contaminants adsorbed from the surrounding water (e.g. polychlorinated biphenyls) [8]–[11]. These substances can be carried across marine regions and transferred from plastics to a wide range of organisms, from zooplankton and small fish to whales [8], [12]–[19]. Furthermore, they can physically damage suspension- and deposit-feeding fauna (e.g. internal abrasions and blockages after ingestion) [20], and alter pelagic and sediment-dwelling biota by modifying physical properties of their habitats [21]. Finally, these small marine plastics can transport rafting species [22]–[27], potentially changing their natural ranges to become non-native species and even invasive pests.

Apart from providing long-lasting buoyant substrata that allow many organisms to widely disperse [28]–[38], marine plastics may also supply energy for microbiota capable of biodegrading polymers and/or associated compounds [27], [39]–[43], and perhaps for invertebrates capable of grazing upon plastic inhabitants. The hydrophobic nature of plastic surfaces stimulates rapid formation of biofilm, which drives succession of other micro- and macro-organisms. This ‘epiplastic’ community appears to influence the fate of marine plastic pollution by affecting the degradation rate [27], [44], buoyancy [3], [45], [46], and toxicity level [43] of plastics. Moreover, epiplastic microbiota could have impacts on the microflora of its consumers, and infectious organisms may reach their hosts through plastic ingestion [27], [43], [47].

Although epiplastic organisms may play an important role in determining the fate and ecological impacts of plastic pollution, little research has been directed to such study, particularly on the inhabitants of the widely dispersed and abundant millimeter-sized marine plastics [43]. In 1972, two papers first reported the occurrence of organisms (diatoms, hydroids, and bacteria) on small plastics (0.1–5 mm long) collected by plankton nets [22], [23]. Further at-sea studies focusing on microplastic fouling biota only emerged in the 2000’s [21], [27], [48]. Zettler et al. (2013) conducted the first comprehensive characterisation of epiplastic microbial communities, which they coined the “Plastisphere” [27]. These authors used scanning electron microscopy (SEM) and next-generation sequencing to analyze three polyethylene and three polypropylene plastic pieces (approx. 2–20 mm long) from offshore waters of the North Atlantic. This pioneer study revealed a unique, diverse, and complex microbial community that included diatoms, ciliates, and bacteria.

**Figure 1:**
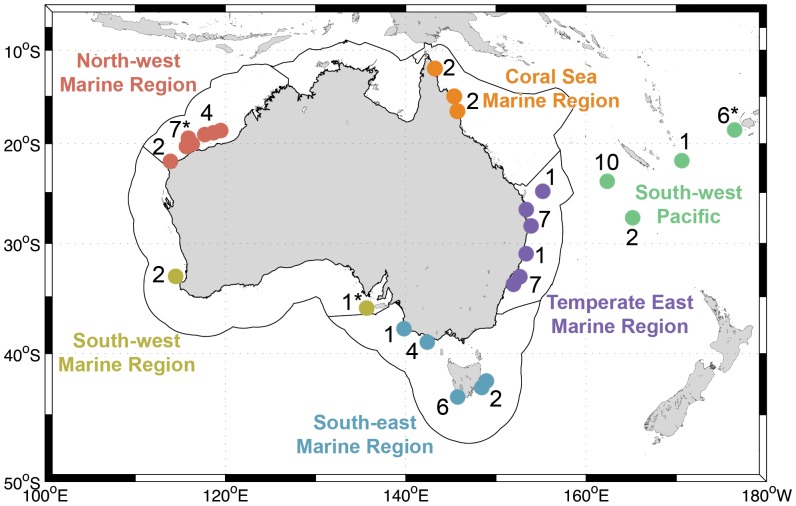
Sampling locations of the 68 plastics analyzed in this study. Black lines delimit marine regions of Australia (environment.gov.au/topics/marine/marine-bioregional-plans); dots indicate areas where the analyzed plastics were collected; numbers represent how many plastics were taken for scanning electron microscopy analyses at these locations. Samples collected were fragments of hard plastic (N = 65), except at locations marked with an asterisk: one piece of Styrofoam cup in Fijian waters, one pellet in South Australia, and one piece of soft plastic in the Australia’s North-west marine region.

Here, we used SEM to examine types of organisms inhabiting the surface of 68 small marine plastics (length range = 1.7–24.3 mm, median = 3.2 mm) from inshore and offshore waters from around the Australian continent (Figure 1). We contributed many new records of taxa associated with millimeter-sized marine plastics and imaged a variety of marine plastic shapes and surface textures resulting from the interaction of polymers with environments and organisms.

## Materials and Methods

Ethics Statement: Permits to conduct field research within the Great Barrier Reef area were obtained from the Great Barrier Reef Marine Park Authority (GBRMPA: permit G11/34378.1). No other special permit was required since sampling was limited to marine debris.

Buoyant plastics were collected using surface net tows in waters around Australia (see details in [4], [49]) and preserved in 2.5% glutaraldehyde buffered in filtered seawater. Prior to analysis with a scanning electron microscope, plastics were dehydrated through a series of increasing ethanol concentrations (up to 100%), critical-point dried using CO_2_, mounted on aluminum stubs with carbon tape, and sputter coated with a 20–30 nm layer of gold. We used a Zeiss 1555 VP-FESEM scanning electron microscope operated at 10–20 kV, 11–39 mm working distance, and 10–30 µm aperture. We randomly selected 65 hard plastics among those small enough to fit onto SEM stubs (<10 mm) and large enough to be easily handled (>1 mm). For comparison, a piece of (1) soft plastic, (2) industrial plastic pellet, and (3) expanded polystyrene (Styrofoam) were also examined, totaling 68 plastic pieces examined with SEM. These plastics were collected from offshore waters of the South-west Pacific (N = 19) and from different Australian marine regions (environment.gov.au/topics/marine/marine-bioregional-plans): North-west (N = 13), South-west (N = 3), South-east (N = 13), Temperate East (N = 16), and Coral Sea (N = 4; Figure 1).

The different types of organisms detected on each plastic piece were imaged, measured using ImageJ (length and width, http://rsb.info.nih.gov/ij/), classified into taxonomic/morphological groups, and the frequency of occurrence (FO) for each type was calculated. We used online resources (e.g. marinespecies.org, westerndiatoms.colorado.edu), primary taxonomic literature (e.g [50]–[54]), and expert consultation (see acknowledgments section) to identify the organisms at the lowest possible taxonomic level. Long filaments were very common but were excluded from the analysis due to difficulty in determining if they were organisms or mucilage.

For each plastic piece observed, an image of the entire piece was taken at 50×magnification. These images were uploaded to ImageJ to measure plastic particles’ size parameters (length, area, perimeter, aspect ratio) and shape parameters (circularity and solidity indexes [55], [56]). Surface fractures, pits and grooves [57], [58] were also observed, recorded, and imaged while examining the entire surface of the plastics at magnifications of 100–500×. Other peculiar microtextures observed at higher magnifications, such as those suggesting interactions with biota, were also recorded and imaged. After SEM analyses, plastics were washed with distilled water and submitted to Fourier Transform Infrared spectrometry (FT-IR) for polymer identification. Two plastic pieces were destroyed while being cleaned for FT-IR; as such, we identified the polymer of 66 out of the 68 plastics examined using SEM.

## Results

**Figure 2:**
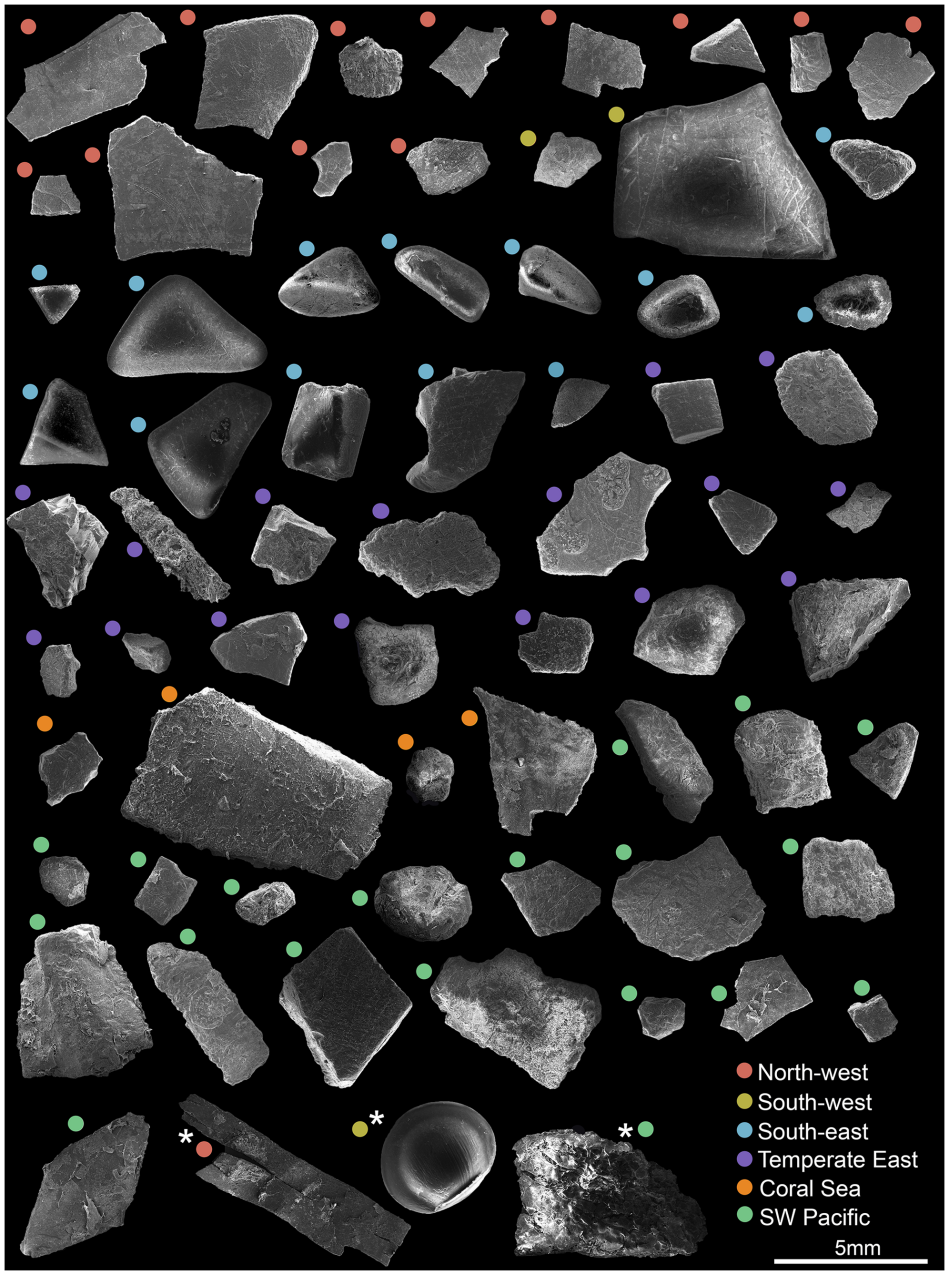
Overall appearance of marine plastics, as shown by scanning electron micrographs. Dot color indicates the marine region where the piece was sampled (see legend and Figure 1). Pieces are hard plastic fragments, with the exception of the soft plastic fragment (red dot), pellet (yellow dot), and Styrofoam fragment (green dot) shown at the bottom of the diagram and marked with a white asterisk. All images are at the same magnification (see scale bar at lower right).

**Figure 3:**
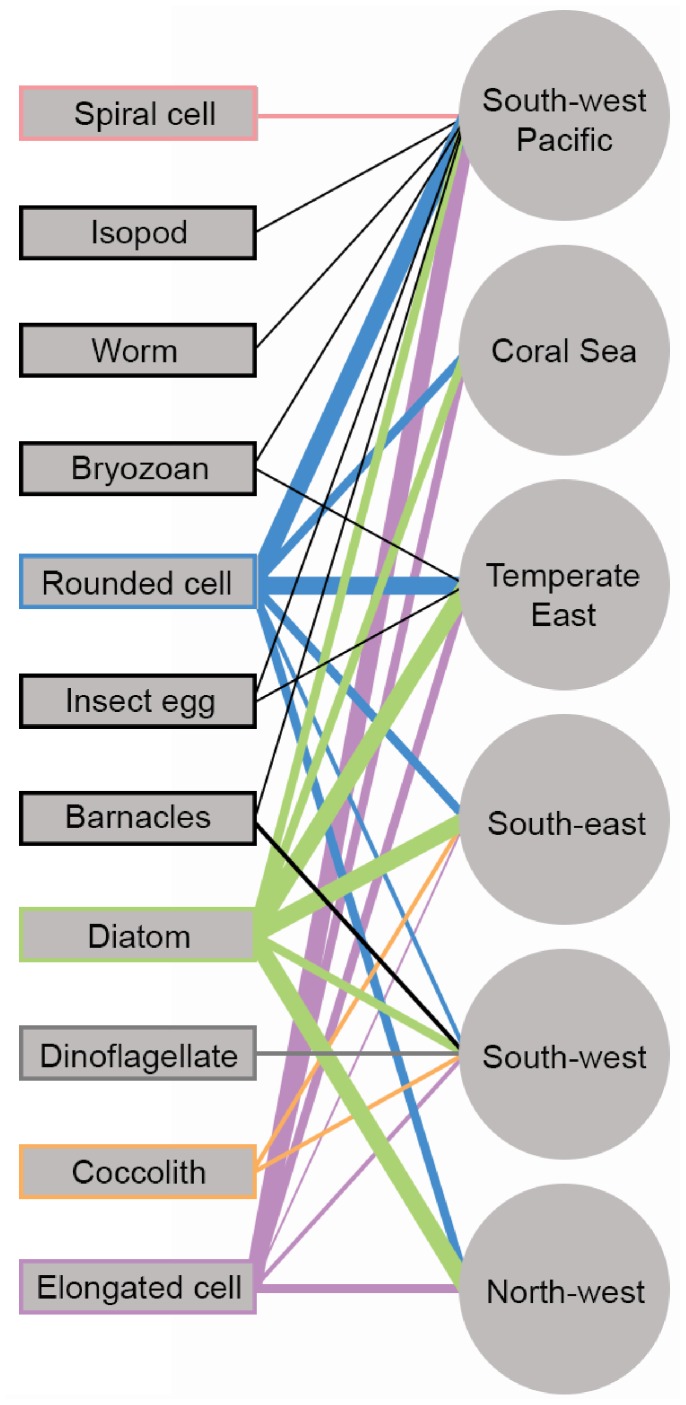
Types of epiplastic organisms detected at each of the marine regions sampled in this study (see **Figure 1**). Lines connect types of organisms (squares) to the marine regions (circles) where they were observed. Line color indicates type of organism, with black lines representing invertebrates. Line thickness is proportional to the organism’s frequency of occurrence (FO = <25%, 25–50%, 50–75%, >75%).

We examined 65 hard plastic fragments with lengths ranging from 1.7 to 8.9 mm (median = 3.2 mm), one 4 mm-wide plastic pellet, one 8.7 mm portion of a 15 mm long soft plastic fragment, and one 7 mm piece of a 24.3 mm Styrofoam cup fragment. Apart from the Styrofoam cup fragment (expanded polystyrene), plastics were made of polyethylene (N = 54) and polypropylene (N = 11). Hard plastics had a diverse range of shapes (solidity index = 0.87–0.98, circularity index = 0.28–0.83; Figure 2) and types of surface microtextures, including linear fractures, pits, and scraping marks (Figure S1). Diatoms and bacteria (rounded, and elongated cells) were by far the most frequently observed organisms, being detected in all sampled marine regions (Figure 3). Plastics’ FT-IR spectra, 1143 SEM micrographs, and a matrix containing information from collection sites, plastics characteristics, and organism/microtexture presence-absence data are available in [59].

**Figure 4:**
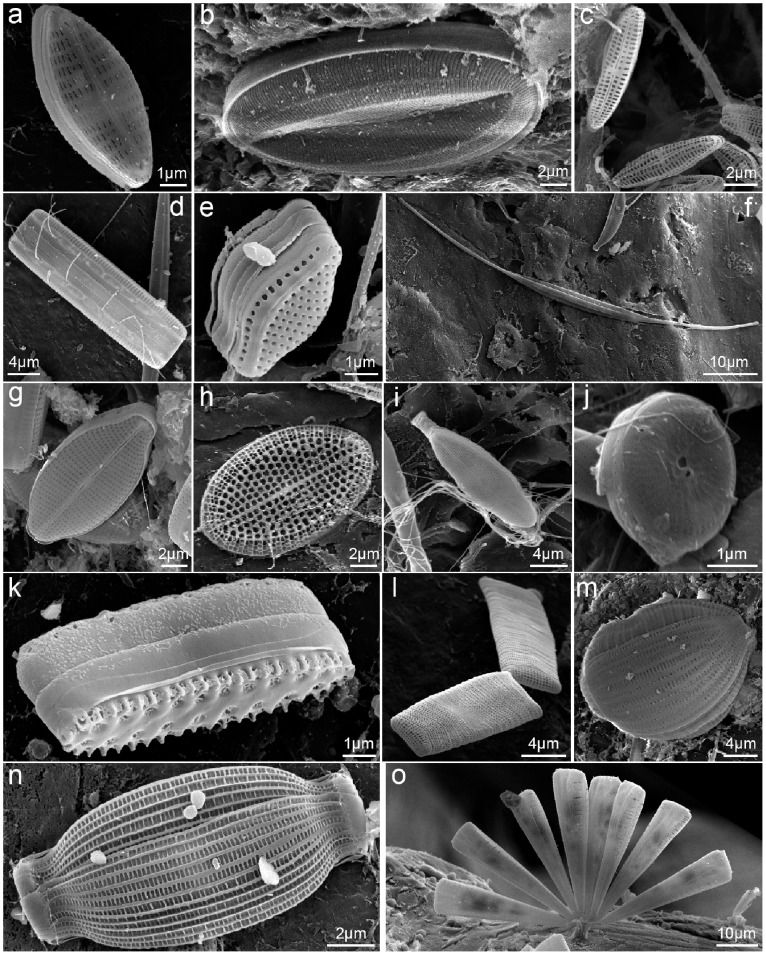
Examples of epiplastic diatoms. a: *Navicula* sp.; b: *Mastogloia* sp.; c: small naviculoids; d: *Nitzschia* sp.; e: *Nitzschia* sp.; f: *Nitzschia longissima*.; g: *Cocconeis* sp.; h: *Cocconeis* sp.; i: *Achnanthes* sp.; j: *Thalassiosira* sp.; k: *Thalassionema nitzschioides*; l: *Microtabella* sp.; m: *Amphora* sp.; n: *Amphora* sp.; o: *Licmophora* sp.

Diatoms were the most abundant, widespread, and diverse group of plastic colonizers (Figures 3 and 4). These organisms were frequently observed (FO = 78%, N = 68 plastics) and included symmetrical biraphids/naviculoids (*Navicula* subgroup lineatae, *Mastogloia* sp., *Haslea* sp.; Figure 4a–c), Nitzschioids (*Nitzschia* spp., *Nitzschia longissima*; Figure 4d–f), monoraphids (*Cocconeis* spp., *Achnanthes* sp.; Figure 4g–i), centrics (*Minidiscus trioculatus*, *Thalassiosira* sp.; Figure 4j), araphids (*Thalassionema nitzschioides* var. *parva*, *Microtabella* spp., *Licmophora* spp., *Grammatophora* sp.; Figure 4k,l,o), and asymmetrical biraphids (*Amphora* spp., *Cymbella* sp.; Figure 4m,n). Most diatoms were growing flat on the surface (adnate and motile diatoms), but some were erect, attached to plastics by mucous pads or stalks/peduncles. The genus *Nitzschia* was the most frequent diatom (FO = 42.6%), followed by *Amphora* (13.2%), *Licmophora* (11.8%), *Navicula* (8.8%), *Microtabella* (5.9%), *Cocconeis* (4.4%), *Thalassionema* (2.9%), and *Minidiscus* (2.9%). The other six genera were only detected on a single plastic piece (FO = 1.5%). These frequencies of occurrence are likely to be underestimated, as many diatoms could not be identified from girdle-view positions (FO unidentified diatoms = 45.6%).

**Figure 5:**
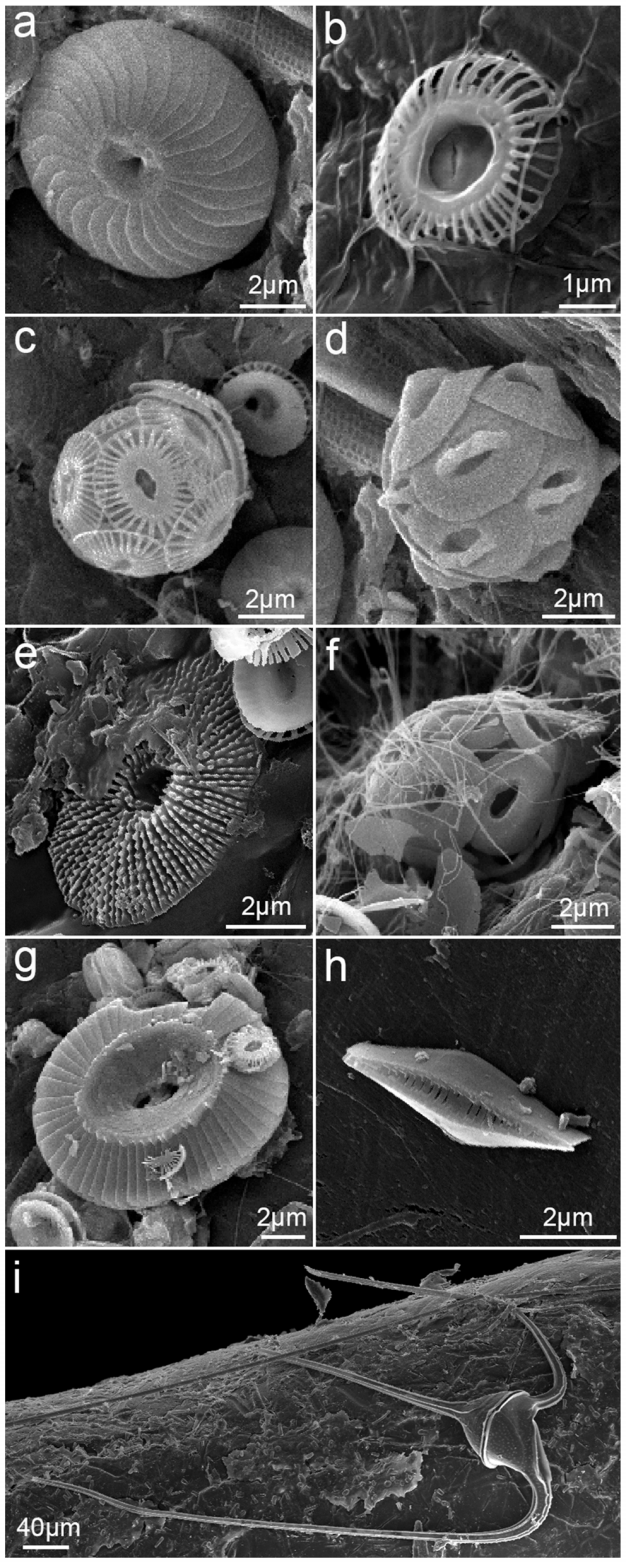
Examples of epiplastic coccoliths and dinoflagellate. a: Calcidiscus leptoporus; b, c: Emiliania huxleyi; d: Gephyrocapsa oceanica; e: Umbellosphaera tenuis; f: Umbilicosphaera hulburtiana; g: Coccolithus pelagicus; h: Calciosolenia sp.; i: Ceratium cf. macroceros dinoflagellate.

Calcareous coccolithophores were observed only on plastics from southern Australia (South-east and South-west marine regions; FO = 37.5%, N = 16 plastics; Figure 3, Figure 5a–h). The species identified included *Calcidiscus leptoporus* (Figure 5a), *Emiliania huxleyi* (Figure 5b,c), *Gephyrocapsa oceanica* (Figure 5d), *Umbellosphaera tenuis* (Figure 5e), *Umbilicosphaera hulburtiana* (Figure 5f), *Coccolithus pelagicus* (Figure 5g), and *Calciosolenia* sp. (Figure 5h). Many of these observations related to detached coccolith scales held in place by mucilage and chitin filaments resembling those produced by diatoms (e.g. *Thalassiosira*; Figure 5b,f). However, intact coccospheres were also present (Figure 5c,d,f). Additionally, one specimen of the dinoflagellate *Ceratium* cf. *macroceros* was present on a 8.2 mm plastic from South-west Australia (Figure 3, Figure 5i).

**Figure 6:**
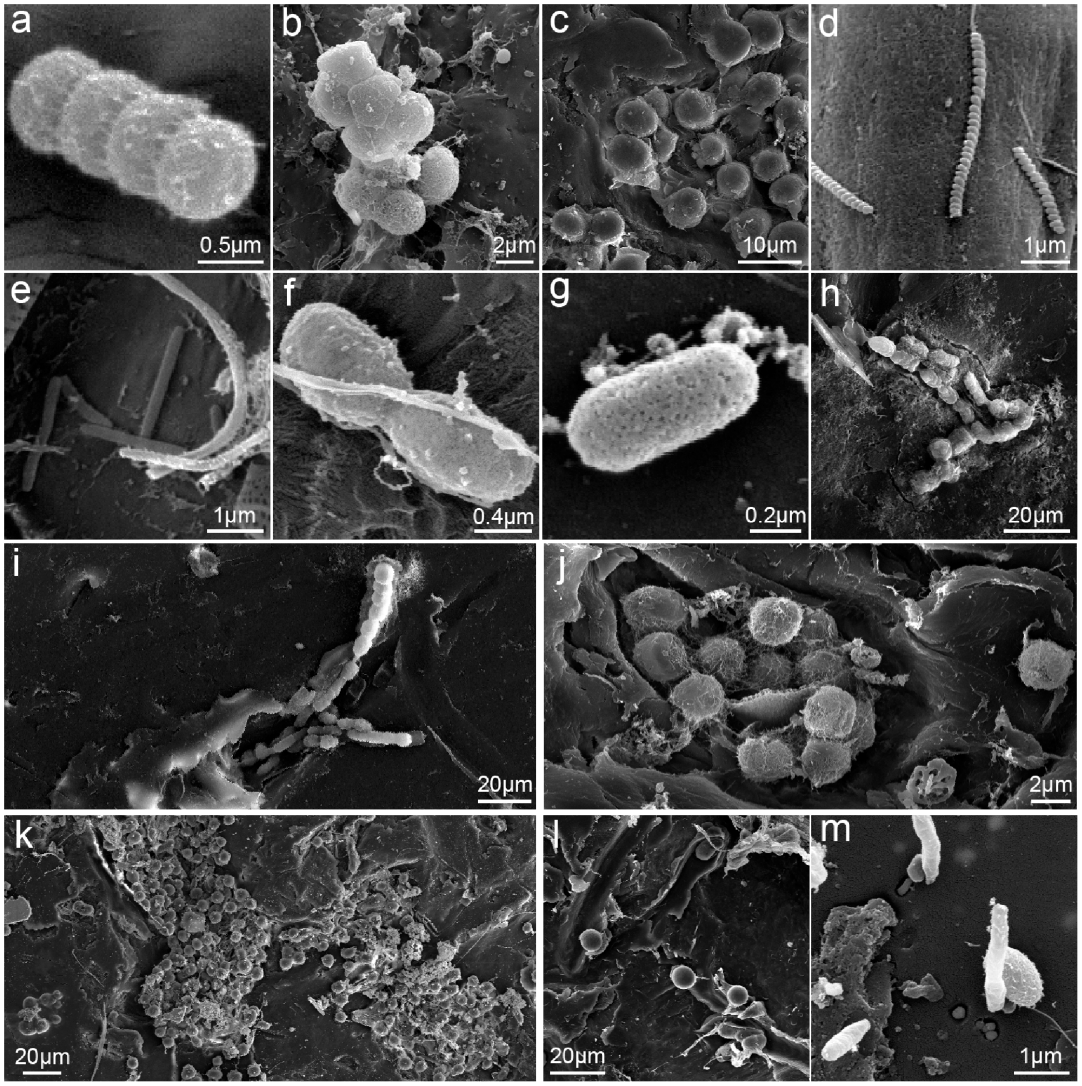
Examples of epiplastic rounded, elongated and spiral cells. a, b, c: rounded cells; d: spiral “spirochaete” cell; e, f, g, h: elongated cells.; i, j, k, l, m: pits and grooves on plastics with rounded cells.

We found several unidentified organisms of various morphotypes and sizes, mostly resembling bacterial, cyanobacterial, and fungal cells (Figure 6). After diatoms, rounded/oval cells (length-width ratio <1.5; Figure 6a–c,i–m) were the most frequently observed morphotype (FO = 72%, N = 68 plastics; Figure 3). Rounded/oval cells with widths <1 µm and ≥1 µm had an overall FO of 38.2% and 54.4%, respectively. Elongated cells (length-width ratio ≥1.5; Figure 6e–h) were also frequently observed, being detected on 59% of the plastics examined (Figure 3). Those with widths <1 µm and ≥1 µm had an overall FO of 51.5% and 11.7%, respectively. Spiral cells (Figure 6d) had similar appearances (resembling spirochaete bacteria) and sizes (0.2–0.3 µm width), and were only observed in the South-west Pacific region (FO = 31.6%, N = 19; Figure 3). Several plastic pits and grooves contained bacteria-like cells closely resembling their shape (Figure 6i–m). They were particularly common on plastics covered by large rounded cells (Figure 6k).

**Figure 7:**
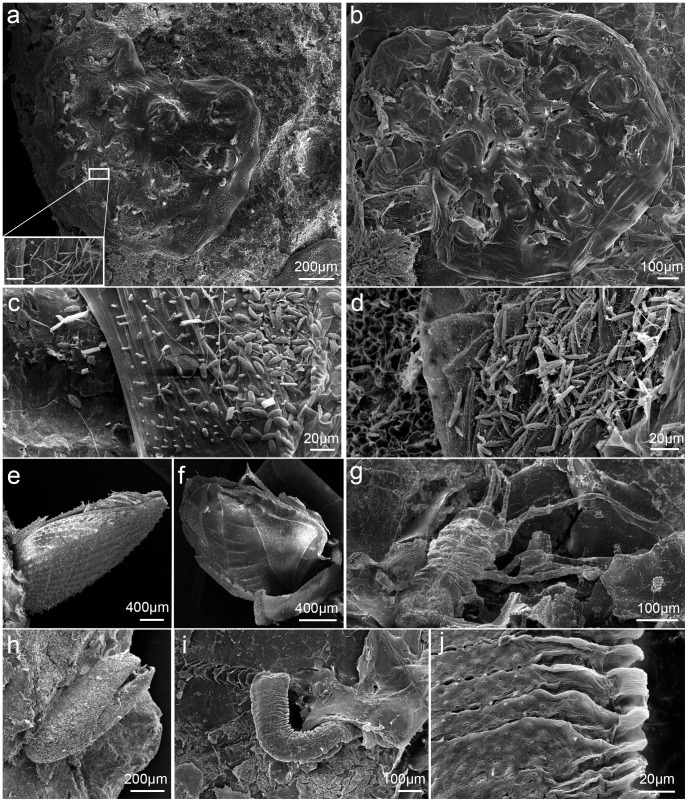
Examples of epiplastic invertebrates. a: Bryozoan colony harboring an abundant assemblage of *Nitzschia longissima* (zoomed image shows part of this assemblage, scale bar = 20 µm); b: bryozoan colony relatively free of fouling; c: bryozoan-plastic interface displaying an abundant epizoic assemblage of *Amphora* sp.; d: bryozoan-plastic interface displaying an abundant epizoic assemblage of *Nitzschia* sp.; e, f: barnacles (*Lepas* spp.); g: Asellota isopod; h: egg of the marine insect *Halobates* sp.; i: marine worm; j: zoom on the surface of the unidentified marine worm.

A few invertebrates were observed on the millimeter-sized plastics (FO = 16.2%, N = 68 plastics; Figures 3 and 7). Colonies of encrusting bryozoans were the most common epiplastic animal (FO = 8.8%; Figure 7a–d). They occurred on two fragments from the Temperate East marine region and on four fragments from oceanic waters of the South-west Pacific (plastic length = 3.2–5.4 mm). Four of these bryozoan colonies were hosting abundant diatom assemblages dominated by *Licmophora* sp., *Nitzschia longissima* (Figure 7a), *Amphora* sp. (Figure 7c), and *Nitzschia* sp. (Figure 7d). Additionally, lepadomorph barnacles (*Lepas* spp.; Figure 7e,f) were attached to the 24.3 mm Styrofoam cup fragment and to a 8.2 mm-long hard plastic; an Asellote isopod (Figure 7g) was found on the Styrofoam cup fragment; eggs of the marine insect *Halobates* sp. (Figure 7h) were observed on two plastics (4.6 and 5.5 mm long); and a unidentified marine worm (Figure 7i,j) was found on a 6 mm hard plastic fragment.

## Discussion

There now exists a large body of evidence that millimeter-sized plastics are abundant and widespread in marine environments [1]–[6], [22], [23] and our study significantly adds to this by conclusively demonstrating that they are colonized by a wide range of biota, particularly diatom and bacteria species (Table 1, [3], [22], [24]–[27]). We more than doubled the number of known diatom genera inhabiting millimeter-sized marine plastics and provide the first identifications of coccolithophore genera attached to these floating plastic particles. We also recorded a few invertebrate species living on these small plastics. As such, our findings provide further evidence that not only large debris [28]–[38] serve as vehicles for organism dispersal. Abundant ‘microplastics’ are equally providing a new pelagic habitat to many microorganism and a few invertebrate taxa.

**Table 1:** List of known genera occurring on millimeter-sized pelagic plastics.

Group	Abundance/FO	Genera
Bacteria^a^^,^ b ^,^ c ^,^ d ^,^ j	d1833 per mm^−2^	*Acinetobacter* b, *Albidovulum* b, *Alteromonas* b, *Amoebophilus* b, *Bacteriovorax* b,*Bdellovibrio* b, *Blastopirellula* b, *Devosia* b, *Erythrobacter* b, *Filomicrobium* b, *Fulvivirga* b,*Haliscomenobacter* b, *Hellea* b, *Henriciella* b, *Hyphomonas* b, *Idiomarina* b, *Labrenzia* b,*Lewinella* b, *Marinoscillum* b, *Microscilla* b, *Muricauda* b, *Nitrotireductor* b,*Oceaniserpentilla* b, *Parvularcula* b, *Pelagibacter* b, *Phycisphaera* b, *Phormidium* b,*Pleurocapsa* b, *Prochlorococcus* b, *Pseudoalteromonas* b, *Pseudomonas* b, *Psychrobacter* b,*Rhodovulum* b, *Rivularia* b, *Roseovarius* b, *Rubrimonas* b, *Sediminibacterium* b,*Synechococcus* b, *Thalassobius* b, *Thiobios* b, *Tenacibaculum* b, *Thalassobius* b, *Vibrio* b
Diatoms^a^^,^ b ^,^ c ^,^ d ^,^ f	a77.9%^d^ 1188 per mm^−2^	***Amphora*** a **, ** ***Achananthes*** a, *Chaetoceros* b, ***Cocconeis*** a, *Cyclotella* c, ***Cymbella*** a,***Grammatophora*** a, ***Haslea*** a, ***Licmophora*** a, *Mastogloia* a ^,^ c, ***Microtabella*** a,***Minidiscus*** a, *Navicula* b, *Nitzschia* a ^,^ b, *Pleurosigma* c, *Sellaphora* b,*Stauroneis* b, ***Thalassionema*** a, ***Thalassiosira*** a
Coccoliths^a^^,^ d ^,^ b	a8.8%	***Calcidiscus*** a **, ** ***Emiliania*** a **, ** ***Gephyrocapsa*** a **, ** ***Umbellosphaera*** a **,** ***Umbilicosphaera*** a **, ** ***Coccolithus*** a **, ** ***Calciosolenia*** a
Bryozoa^a^^,^ e ^,^ f	a8.8%	*Membranipora* f, *Jellyella* e, *Bowerbankia* e, *Filicrisia* e
Hydroids^c^^,^ e	–	*Clytia* c, *Gonothyraea* c, *Obelia* e
Polychaete^g^	–	*Spirorbis* g, *Hydroides* g
Dinoflagellates^a^^,^ b ^,^ d	a1.5%	*Alexandrium* b, *Ceratium* a
Insect eggs^a^^,^ h ^,^ i	a2.9%	*Halobates* a ^,^ h ^,^ i
Barnacles^a^	a2.9%	***Lepas*** a
Rhodophyta^b^^,^ g	–	*Fosliella* g
Foraminifera^g^	–	*Discorbis* g
Radiolaria^b^^,^ d	–	*Circorrhegma* d
Ciliate^b^	–	*Ephelota* b

Organism groups (first column), their abundance and/or frequency of occurrence (when available; second column), and genera (third column). References are indicated by superscript letters and given at the bottom of the table, along with approximate length range of plastics examined. Genera in bold indicate those first detected in this study.

a This study (1.7–24.3 mm),

b Zettler et al. 2013 (2–20 mm) [27],

c Carpenter and Smith 1972 (2.5–5 mm) [22],

d Carson et al. 2013 (1–10 mm) [26],

e Goldstein et al. 2014 (4–5 mm) [38],

f Gregory 1978 (2–5 mm) [24],

g Gregory 1983 (1–5 mm) [25],

h Majer et al. 2012 (2–5 mm) [74],

i Goldstein et al. 2012 (1.2–6.5 mm) [48],

j Carpenter et al. 1972 (0.1–2 mm) [23].

We observed fouling diatoms to be diverse and widespread on marine plastics. These diatoms seemed to firmly attach to the plastic, resisting water turbulence and wave action. All the identified diatom genera are well known to form biofilms on estuarine and marine sediments and rocks (epilithic), vegetation (epiphytic), and animals (epizoic) [60]–[65]; marine plastics thus create a novel, long-lasting and abundant floating habitat for ‘benthic’ diatoms, in a light and nutrient-filled environment that is stable and beneficial to these organisms. Future epiplastic diatom research should focus on the quantitative contribution of these organisms to enhancing primary and secondary productivity of different marine regions, such as within subtropical gyres where productivity tends to be low but plastic pollution level high [1]–[3], [66]. Because of their rapid growth and production of extracellular substances [67], epiplastic diatoms may provide an important food source for invertebrate grazers. As plastic debris can contain harmful substances [8], [10]–[12], [19], it remains unclear if such grazer-plastic relationships would have a positive or negative impact on the populations involved in this new type of food web.

A significant number of coccolithophore species were present on millimeter-sized marine plastics. These planktonic organisms are not commonly recognized as fouling or rafting organisms [36], although their occasional occurrence on marine plastics was briefly mentioned in recent studies [26], [27]. Some of our observations were of clusters of mixed coccolith species, resembling zooplankton fecal pellets, and of solitary coccoliths, likely detached from living coccospheres and stuck to clingy parts of the plastic biofilm. However, entire coccolithophores were also seen attached to plastics, suggesting that these organisms could be using ocean plastics as ‘floating devices’. We only observed coccoliths on plastics from southern Australia; as such, additional studies in these temperate waters may help better understand this potential coccolith-plastic relationship. Another atypical organism detected was the planktonic dinoflagellate *Ceratium* cf. *macroceros.* Recent studies have found plastics heavily fouled by dinoflagellates, including individuals and cysts of the potentially harmful species *Ostreopsis* sp., *Coolia* sp., and *Alexandrium* spp. [27], [31], but here we only detected a single specimen of this group.

Several unidentified organisms (rounded, oval, elongated, and spiral) resembling bacterial cells were flourishing on millimeter-sized marine plastics. This supports previous studies that describe well established bacterial populations growing on plastic fragments [26], [27]. Many of these unidentified cells were apparently interacting with the plastic surface by forming pits and grooves. Within this group of “pit-formers”, colonies of rounded cells (around 5 micron in diameter) covered large areas of the plastic surface. They were similar to some previously unidentified epiplastic organisms from the North Atlantic [27]. These SEM observations, along with detections of hydrocarbon-degrading bacteria genes on marine plastics [27] and experiments demonstrating that marine bacteria can biodegrade polymers [27], [39]–[43], strongly suggest that plastic biodegradation is occurring at the sea surface. Such process could partially explain why quantities of millimeter-sized marine plastics are not increasing as much as expected [2], [7]. Studies of the “Plastisphere” from different marine regions worldwide will prove invaluable for extending our knowledge on epiplastic marine microbial communities, and may support the development of biotechnological solutions for better plastic waste disposal practices [68]–[70].

A number of invertebrates inhabited the small plastics examined here: bryozoans, barnacles *Lepas* spp., an Asellota isopod, a marine worm, and eggs of the marine insect *Halobates* sp. Even though microplastic-associated animals are rare and less diverse when compared to those associated with macroplastics [28]–[38], ecological implications of this phenomenon may be significant (e.g. [48]), given the large quantities and wide distribution ranges of millimeter-sized plastics in the marine environment [1]–[6], [22], [23]. Among the effects plastic associates may have is to shape ‘epiplastic’ microbiota by hosting unique epizoic assemblages on their bodies. For instance, the bryozoan colonies examined here covered a large proportion of their plastic-host, with some of them harboring unique diatom-dominated assemblages. Previous studies have shown that bryozoans do not represent neutral surfaces for microbial colonizers [71], [72], with some species offering a favorable habitat for diatoms when compared to the surrounding substratum (e.g. by protecting against predators and supplying nutrients through flow generated by polypids [73]). Further studies focusing on both epiplastic microorganisms and invertebrates have the potential to further elucidate symbiotic and/or competitive relationships between inhabitants of this new type of pelagic habitat.

In summary, this study showed that millimeter-sized marine plastics are providing a new niche for several types of microorganisms and some invertebrates. This phenomenon has considerable ecological ramifications and deserves further research. As discussed here, additional observational and experimental studies on the inhabitants of these small plastic fragments may better elucidate several key plastic pollution processes that remain poorly assessed, such as at-sea polymer degradation and mineralisation, impacts of epiplastic communities on their consumers, and changes in the distributional range of species by plastic rafting.

## Supporting Information

Figure S1 **Examples of marine plastics’ surface textures.** a, d: polypropylene plastics with linear fractures and pits; b, c: higher magnification of the plastic surface shown in ‘a’ (note very similar pits – one empty and one with a cell conforming its shape); e: higher magnification of the plastic surface shown in ‘d’ (note three equally spaced deep pits); f: polyethylene soft plastic with linear fractures, producing squared microplastics; g: higher magnification of the plastic surface shown in ‘f’ (note shallow pits likely formed by *Cocconeis* sp.); h: rounded scrape mark similar to the ones found close to the worm-like animal (see Figure 6i); i,k: sub-parallel scrape marks; j: large plastic pit likely formed by an egg of *Halobates* sp.
(TIF)
