# Supplementary figures and images for: Millimeter-Sized Marine Plastics: A New Pelagic Habitat for Microorganisms and Invertebrates
Source: PLoS One. 2014 Jun 18;9(6):e100289. doi: 10.1371/journal.pone.0100289 (PMC4062529; doi:10.1371/journal.pone.0100289)

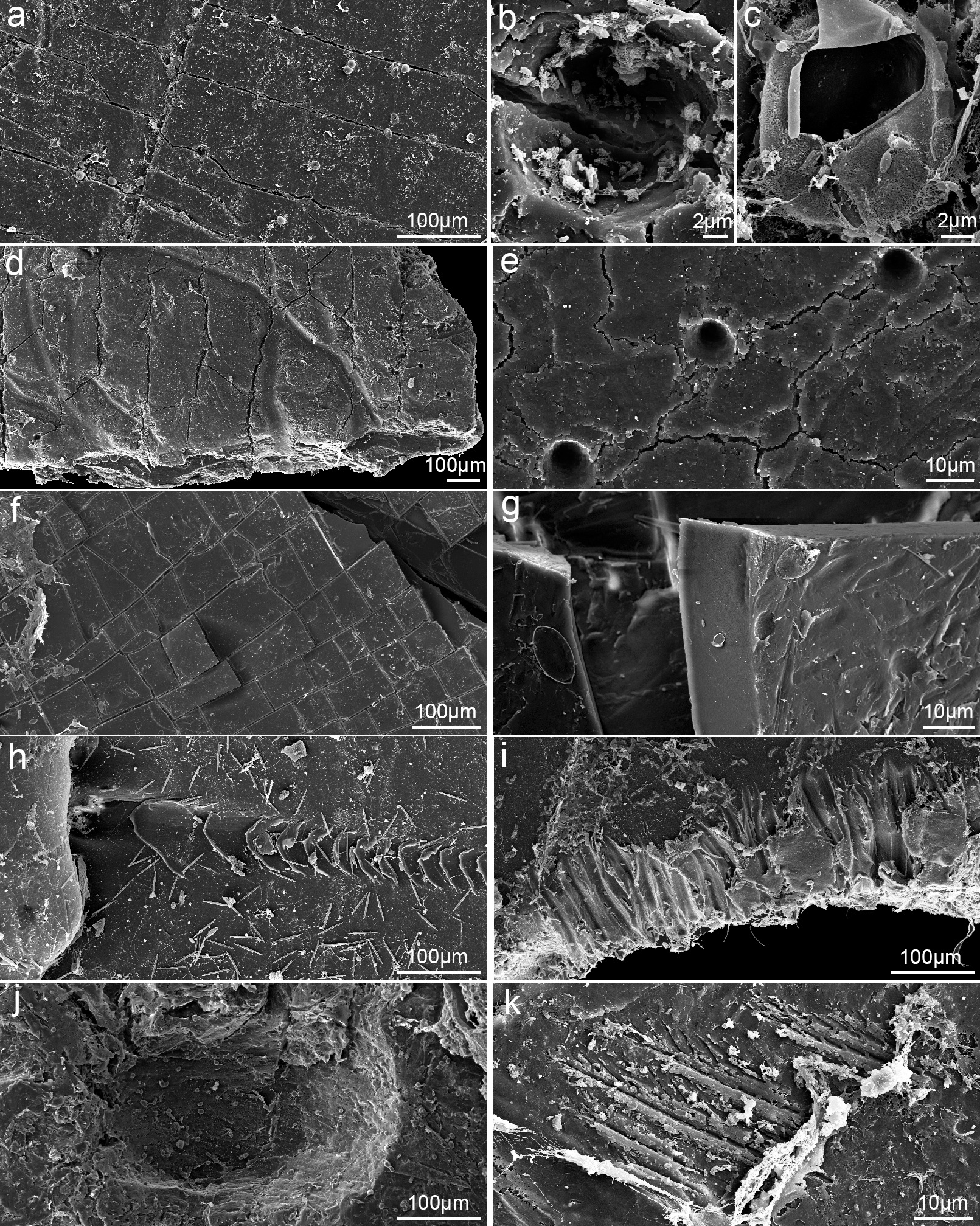

Supplement: Figure S1 — Examples of marine plastics’ surface textures. a, d: polypropylene plastics with linear fractures and pits; b, c: higher magnification of the plastic surface shown in ‘a’ (note very similar pits – one empty and one with a cell conforming its shape); e: higher magnification of the plastic surface shown in ‘d’ (note three equally spaced deep pits); f: polyethylene soft plastic with linear fractures, producing squared microplastics; g: higher magnification of the plastic surface shown in ‘f’ (note shallow pits likely formed by Cocconeis sp.); h: rounded scrape mark similar to the ones found close to the worm-like animal (see Figure 6i); i,k: sub-parallel scrape marks; j: large plastic pit likely formed by an egg of Halobates sp. (TIF) [file pone.0100289.s001.tif]
